# Editorial: Innovative models of stroke pathology

**DOI:** 10.3389/fneur.2023.1266075

**Published:** 2023-08-04

**Authors:** Siobhan Crilly, Marietta Zille, Paul R. Kasher, Michel Modo

**Affiliations:** ^1^Division of Neuroscience, School of Biological Sciences, Faculty of Biology, Medicine and Health, Manchester Academic Health Science Centre, The University of Manchester, Manchester, United Kingdom; ^2^Geoffrey Jefferson Brain Research Centre, Manchester Academic Health Science Centre, Northern Care Alliance and The University of Manchester, Manchester, United Kingdom; ^3^Department of Pharmaceutical Sciences, Division of Pharmacology and Toxicology, University of Vienna, Vienna, Austria; ^4^Department of Radiology, University of Pittsburgh, Pittsburgh, PA, United States

**Keywords:** stroke, pre-clinical, clinical research, ischemic stroke, brain hemorrhage

Despite our best attempts to understand and treat the disease, stroke remains a leading cause of death and disability worldwide. Both ischemic and hemorrhagic strokes cause a significant global health burden and therefore innovative insights into modeling disease and subsequent pathology are imperative to elucidating the complexities and identify therapeutic strategies. In this Research Topic, we have assembled studies that address this need for innovation to provide novel approaches and analyses that advance both clinical and pre-clinical modeling of stroke.

Historically, the focus of pre-clinical research has primarily been to elucidate disease pathology and identify drugs for translational treatment. Occasionally, re-evaluating the standard models and approaches can lead to greater advances. Pinto et al. have characterized the optimal filament and occlusion time for the middle cerebral artery occlusion (MCAO) model in mice to improve translational outcomes in terms of lesion volume. Through applying a reverse translational post-operative care strategy, as is recommended for stroke patients, the authors have increased the relevance of their results while including pain control as well as an improved ease of access to food and fluid supplementation for the mice. Often in the literature, MCAO approaches vary greatly and so adoption of the authors' optimized approach will aim to increase the reproducibility and translatability of results. von Seckendorff et al. used an *in vitro* simulator to demonstrate that the length and accuracy of endovascular therapy (EVT) is influenced by the composition of the thrombus. EVT is the gold-standard therapy for ischemic stroke patients however it is only beneficial in 25% of cases. A “back to the bench” approach to investigate thrombus composition to inform clinical practice may improve recanalization efficacy and reduce the risk of embolization to the patient.

Large mammalian models offer certain advantages over rodents, namely the increased size of white matter with a gyrencephalic brain. Sorby-Adams et al. employed a novel approach to study gait kinematics in an ovine model of ischemic stroke, addressing the need for functional assessments in large mammals. Using infrared cameras and motion capture technology, the authors determined that their approach was effective at detecting acute functional changes, demonstrating for the first time, an effective analysis that can be applied to large mammalian models. Ye et al. developed a novel model of MCAO-generated ischemic stroke in cynomolgus monkeys with autologous thrombi. The authors demonstrated reproducible strokes and similar pathology to clinical outcomes. Non-human primates (NHPs) are the closest animal model to humans, possessing 90–93% genetic similarity, larger brains and similar cerebrovascular morphology. They are also the closest representative for studies pertaining to stroke co-morbidities. Both of these larger mammalian models offer advantages over rodents, such as the ability to investigate white matter changes in a gyrencephalic brain and to use clinical stroke scales in NHPs.

Clinical treatment of stroke patients is limited to surgical intervention where possible (EVT and hematoma aspiration) and specialized hospital care. The majority of stroke patients are likely of advanced age and receiving medications for co-morbid conditions e.g., hypertension and rarely exhibit stroke in isolation. Ding et al. have developed a clinical tool based on the NIH stroke scale score, age and stroke history to predict outcomes at 3 months. Such a predictive tool may have direct use when designing treatment plans for individual patients, and provide a reference basis for determining interventional outcome. Considering that pre-clinical modeling of co-morbidities is incredibly difficult, Yu-Huan et al. have piloted a retrospective clinical study to determine why patients receiving hemodialysis are at increased risk of suffering hemorrhagic stroke. The results determined that higher blood pressure and abnormal intact parathyroid hormone metabolism contribute to increased risk. Consideration of these factors in patients on hemodialysis might prove preventative for ICH. Additionally, understanding the role of anti-coagulant choice in dialysis patients on worsening stroke outcomes might lead to personalized management methods.

Our intention for this Research Topic is to highlight new and novel approaches to stroke modeling, so that both clinical and pre-clinical researchers can benefit from these approaches to improve the drug discovery pipeline and patient management.

## Author contributions

SC: Writing—original draft, Writing—review and editing. MZ: Writing—review and editing. PRK: Writing—review and editing. MM: Writing—review and editing.

